# Haemophilus influenzae Serotype a Invasive Disease, Alaska, USA, 1983–2011

**DOI:** 10.3201/eid1906.121805

**Published:** 2013-06

**Authors:** Michael G. Bruce, Tammy Zulz, Carolynn DeByle, Ros Singleton, Debby Hurlburt, Dana Bruden, Karen Rudolph, Thomas Hennessy, Joseph Klejka, Jay D. Wenger

**Affiliations:** Centers for Disease Control and Prevention, Anchorage, Alaska, USA (M.G. Bruce, T. Zulz, C. DeByle, D. Hurlburt, D. Bruden, K. Rudolph, T. Hennessy, J.D. Wenger);; Alaska Native Medical Center, Anchorage (R. Singleton);; Yukon Kuskokwim Health Corporation, Bethel, Alaska (J. Klejka)

**Keywords:** *Haemophilus influenzae*, *H. influenzae*, Hia, Hib, bacteria, coccobacillus, Alaska, United States, Alaska Native people, invasive disease, pneumonia, bacteremia, meningitis, epiglottitis, septic arthritis, cellulitis, otitis media, pericarditis

## Abstract

Before introduction of *Haemophilus influenzae* type b (Hib) vaccines, rates of Hib disease in Alaska’s indigenous people were among the highest in the world. Vaccination reduced rates dramatically; however, invasive *H. influenzae* type a (Hia) disease has emerged. Cases of invasive disease were identified through Alaska statewide surveillance during1983–2011. Of 866 isolates analyzed for serotype, 32 (4%) were Hia. No Hia disease was identified before 2002; 32 cases occurred during 2002–2011 (p<0.001). Median age of case-patients was 0.7 years; 3 infants died. Incidence of Hia infection (2002–2011) among children <5 years was 5.4/100,000; 27 cases occurred in Alaska Native children (18/100,000) versus 2 cases in non-Native children (0.5/100,000) (risk ratio = 36, p<0.001). From 12/2009 to 12/2011, 15 cases of Hia disease occurred in southwestern Alaska (in children <5 years, rate = 204/100,000). Since introduction of the Hib conjugate vaccine, Hia infection has become a major invasive bacterial disease in Alaska Native children.

*Haemophilus influenzae* is a bacterial pathogen that can cause serious invasive disease. The organism is classified by the presence of a capsular polysaccharide (6 STs, a-f) or its absence (nonencapsulated or nontypeable strains). In Alaska, before introduction of *H. influenzae* serotype b (Hib) vaccine, rates of invasive Hib disease among Alaska Native people were among the highest in the world (*1*,*2*). Disease caused by Hib was reduced significantly after the 1991 introduction of the Hib conjugate vaccine, polyribosylribitol phosphate outer membrane protein (PRP-OMP) (*3*); however, this vaccine does not provide protection against the other capsular or nontypeable strains (*4*). *H. influenzae* serotype a (Hia), in particular, has been reported as the cause of serious disease in young children (*5**–**7*). We previously described invasive Hia disease in the North American Arctic (Northern Canada and Alaska) from 2000 through 2005 (*8*) and reported an outbreak of 5 episodes of invasive Hia disease in 3 children during 2003 in southwestern Alaska (*9*). Sporadic cases were reported from 2005 through 2009. In this report, we describe an outbreak of 15 cases of invasive Hia disease that occurred from December 2009 through December 2011 in neighboring areas and review the microbiology and epidemiology of Hia disease in Alaska from 1983 to 2011.

## Methods

Statewide surveillance for all invasive *H. influenzae* disease in Alaska has been conducted since 1980 by the Centers for Disease Control’s Arctic Investigations Program (AIP) based in Anchorage, Alaska. Clinical laboratories are requested to send *H. influenzae* isolates recovered from a normally sterile site (e.g., blood, cerebrospinal fluid, pleural fluid, etc.) in a resident of Alaska to AIP for confirmation of identity and serotyping. *H. influenzae* is confirmed by using Gram stain and factor X and V requirements (Differentiation Disks; Difco Laboratories, Detroit, MI, USA). Serotyping is conducted by using slide agglutination (*H. influenzae* types a–f typing antisera; Difco). Since 2005, a small number of *H. influenzae* cases have also been identified by PCR on specimens from patients whose conditions had been treated with antimicrobial drugs before samples were collected and whose samples from sterile sites were culture negative. PCR amplification of serotype-specific genes was done by using primers and probes as reported by Maaroufi, et al. (*10*). Multilocus sequence typing (MLST) of 7 housekeeping gene loci was done on all invasive Hia isolates according to a previously described method (*11*). The sequence type (ST) assignments were made by using the *H. influenzae* MLST website (http://haemophilus.mlst.net/). Clonal complexes were assigned by using the eBURST algorithm with software available at the MLST website (http://www.mlst.net). The IS*1016-*bexA deletion was amplified from genomic DNA by PCR by using sense IS*1016* (5′-ATTAGCAAGTATGCTAGTCTAT-3′) and antisense bexA (5′-CAATGATTCGCGTAAATAATGT-3′)primers (*12*). AIP uses a standardized form to collect demographic and clinical data from medical records for each reported case. Each year, a list of submitted samples is reconciled with culture results from each participating laboratory, and case lists are compared with state-reportable disease data. Serotyping of invasive *H. influenzae* isolates was introduced gradually; the practice grew from serotyping isolates from 8% of collected samples in 1980 to serotyping 80% by 1983. Because serotyping was intermittent from 1980 to 1982, we reviewed data from 1983 to 2011.

A case of invasive *H. influenzae* disease was defined by isolation of *H. influenzae* from a normally sterile site, including blood, cerebrospinal fluid, pleural fluid, peritoneal fluid, or joint fluid from a resident of Alaska. Cases in patients with clinical epiglottitis (who had *H. influenzae* isolated from an epiglottis swab sample) were also reportable. The clinical description of *H. influenzae* infection was determined by a review of the discharge diagnoses in each case-patient’s medical record. When case-patients had multiple discharge diagnoses, the diagnoses related to invasive Hia infection were ranked according to severity, from highest to lowest, in the following order: meningitis, epiglottitis, pneumonia, pericarditis, osteomyelitis, septic arthritis, and septicemia with unknown focus.

We defined an outbreak of invasive Hia disease as the occurrence of confirmed cases of invasive Hia infection by isolates within the same clonal complex among persons residing in the same region with incidence rates above baseline. Population denominator data for Alaska were obtained from the Alaska Department of Labor and Workforce Development website (http://www.labor.state.ak.us). Estimates for calculating rates of Hia disease in Alaska reflect population figures derived from the 2000 and 2010 census counts; rates were calculated for the years 2002–2011. During 2002–2011, Alaska’s population ranged from 640,841 to 722,190; Alaska Native peoples comprised 19% of the population. Seventy-five percent of the population resided in urban centers; the remaining population was dispersed widely across 586,412 square miles. We defined Region A as the southwestern region of Alaska, which has a population of ≈25,000 persons.

## Results

### Descriptive Epidemiology

**Figure 1:**
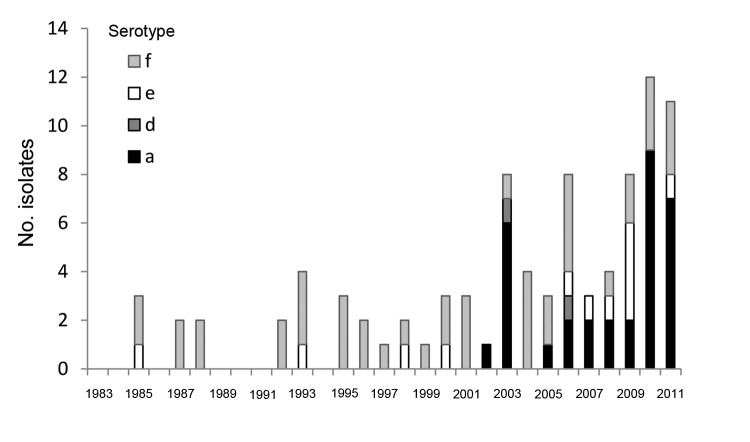
Reported cases of non-b encapsulated *Haemophilus influenza* disease, Alaska, 1983–2011.

We identified 958 cases of invasive *H. influenzae* disease during 1983–2011 (data not shown); of these, 858 isolates were available for serotyping by slide agglutination. Eight additional culture-negative sterile site samples obtained from patients with clinically compatible illnesses who had been treated with antimicrobial drugs were identified, and isolates were serotyped by using PCR. Among samples serotyped, 617 were serotype b and 158 were nontypeable. Of the remaining 91 typeable, non–serotype b isolates, 44 (48%) were serotype f, 32 (35%) were serotype a, 13 (14%) were serotype e, and 2 (2%) were serotype d (Table 1). Hif and Hie isolates were identified from 1985 onward; the first Hia isolate was identified in 2002. During 1983–2001, none of the 30 encapsulated, non–serotype b isolates were Hia; however, from 2002 through 2011, 32 (52%) of 62 non–serotype b isolates were Hia (p<0.001 vs. 1983–2001) (Figure 1). Five (16%) of 32 Hia cases were identified by PCR.

**Table 1:** Typeable and nontypeable *Haemophilus influenzae* isolates, Alaska 1983–2011

Year	Isolate type							
	a	b	c	d	e	f	NT*	Total
1983	0	71	0	0	0	0	4	75
1984	0	73	0	0	0	0	5	78
1985	0	88	0	0	1	2	2	93
1986	0	82	0	0	0	0	3	85
1987	0	52	0	0	0	2	4	58
1988	0	60	0	0	0	2	0	62
1989	0	44	0	0	0	0	1	45
1990	0	44	0	0	0	0	2	46
1991†	0	22	0	0	0	0	3	25
1992	0	3	0	0	0	2	0	5
1993	0	8	0	0	1	3	2	14
1994	0	2	0	0	1	0	11	14
1995	0	4	0	0	0	3	10	17
1996	0	8	0	0	0	2	4	14
1997	0	9	0	0	0	1	6	16
1998	0	5	0	0	1	1	8	15
1999	0	8	0	0	0	1	2	11
2000	0	9	0	0	1	2	3	15
2001	0	1	0	0	0	3	6	10
2002	1	1	0	0	0	0	8	10
2003	6	2	0	1	0	1	8	18
2004	0	2	0	0	0	4	5	11
2005	1	4	0	0	0	2	2	9
2006	2	3	0	1	1	4	7	18
2007	2	0	0	0	1	0	10	13
2008	2	3	0	0	1	1	13	20
2009	2	4	0	0	4	2	8	20
2010	9	3	0	0	0	3	11	26
2011	7	2	0	0	1	3	10	23
Total	32	617	0	2	13	44	158	866

*NT, not typeable. †*Haemophilus influenza type* b conjugate vaccine introduction in Alaska.

### Invasive Hia Cases, 2002–2011

**Figure 2:**
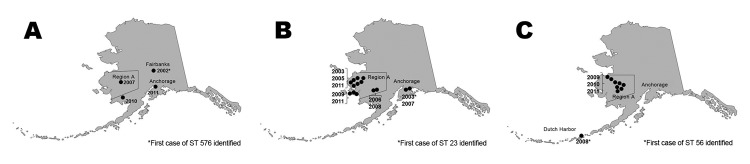
Geographic distribution of invasive *Haemophilus influenzae* type a disease in Alaska, by sequence type (ST). A) ST 576; B) ST 23; C) ST 56.

Among the 32 Hia cases, 28 (88%) occurred in children <2 years of age, 1 (3%) occurred in a child 2–4 years, and 3 (9%) occurred in persons >5 years (range 8–48 years). The median age of case-patients was 0.7 years (range 0.3–48 years). Ethnicity data were available for all cases; 28 (88%) occurred in Alaska Native persons. Cases occurred in 5 areas of Alaska; 21 (66%) cases occurred in Region A (Figure 2). Among all invasive Hia cases, the most common clinical syndromes were meningitis (12 [38%]), pneumonia with bacteremia (11 [34%]), and septic arthritis (6 [19%]). All cases of meningitis occurred in children <2 years of age; there were no cases of epiglottitis. Twenty-seven (84%) case-patients were hospitalized. Three case-patients <1 year of age died (case-fatality ratio 9%). Twenty-five (78%) case-patients <5 years of age had been vaccinated for Hib at appropriate ages. No seasonal pattern among Hia cases was observed. Two distinct outbreaks, 1 in 2003 (5 cases) (*9*) and the other in 2009–2011 (15 cases), comprised 63% of the 32 invasive Hia cases that occurred from 2002–2011. Although there were 20 Hif cases during the same period, the epidemiology differs from Hia cases; Hif cases tend to occur in older persons (median age of case-patients: 55 years), in non–Alaska Native persons (70%), and a higher proportion of case-patients (45%) have a clinical syndrome of pneumonia with bacteremia. 

### Description of the 2009–2011 Hia Outbreak 

From December 1, 2009, through December 31, 2011, 15 cases of Hia occurred in Region A, 10 (67%) of which were identified either in the largest community (4 cases) or in nearby villages (6 cases). Thirteen of the 15 cases occurred in Alaska Native children <2 years of age; 1 was in an Alaska Native child 8 years of age, and 1 was in a non-Native adult, who was 48 years of age. The median age of case-patients was 0.8 years (range 0.3–48 years). Of the 13 cases in Alaska Native children <5 years of age, 8 (62%) were in girls. There was 1 death (case-fatality ratio 8%). Primary clinical conditions included meningitis (n = 6), pneumonia with bacteremia (n = 3), septic arthritis (n = 2), cellulitis (n = 1), and bacteremia (n = 1). Four case-patients had underlying conditions, including a history of otitis media, chronic pulmonary disease, premature birth, esophageal reflux, abnormal heart murmur, or pulmonary valve stenosis, and 1 child experienced a stroke while hospitalized. Of these children, 1 was not vaccinated with PRP-OMP at the appropriate age.

### Incidence Rates

The overall rate of invasive Hia disease in Alaska during 2002–2011 was 5.4/100,000 population (95% CI 3.6–7.7) among children <5 years of age; however, the rate in Alaska Native children <5 years was 36 times higher than in non-Native children (18/100,000 versus 0.5/100,000, p < 0.0001). Overall rates of invasive Hib and Hif disease in children in Alaska <5 years of age during 2002–2011 were 2.8/100,000 (95% CI: 1.6–4.6) and 0.9/100,000 (95% CI: 0.3–2.2), respectively. Invasive Hia disease occurred primarily in Region A, where the rate among children <5 years of age was 72.3/100,000 compared with 1.2/100,000 in children <5 years of age in the rest of the state (p<0.0001). During 2010, the year in which the most cases occurred during the most recent outbreak, the rate of invasive Hia disease in children <5 years of age in Region A was 250/100,000 compared to the rate for previous years of 45/100,000 (p<0.001 Fisher exact test).

### Subtyping Data

MLST of the 27 Hia isolates yielded 3 sequence types (STs), which are shown by month and year (Table 2; Figure 2). eBURST analysis showed that these 3 STs fell into a single clonal complex: CC23. ST576 was identified 4 times during the 26-year surveillance period and is a double locus variant of ST23. Of 14 ST23 isolates, 9 (64%) were found during 2003–2007. All of the ST56 isolates, which are single locus variants of ST23, were found during 2008–2011. Each of these STs first appeared in a port or major city in Alaska and was subsequently isolated from samples from patients in the western rural regions of the state (Figure 2). None of the Hia isolates had evidence of the IS*1016*-bexA partial deletion.

**Table 2:** Sequence type results for *Haemophilus influenzae* type a strains, by date of onset, Alaska, 2002–2011

Year	Month	Sequence type
2002	Nov	576
2003	Jun	23
	Jul	23
	Aug	23
	Oct	23
	Nov	23
	Dec	23
2005	Jul	23
2006	Feb	23
2007	Jul	23
	Oct	576
2008	Apr	23
	Oct	56
2009	Jun	56
	Dec	23
2010	Feb	56
	Jun	56
	Jul	576
	Aug	56
	Oct	56
	Dec	56
2011	Jan	23
	Jan	56
	May	23
	Aug	23
	Sep	576

## Discussion

The 2009–2011 outbreak investigation of invasive Hia disease in Region A, highlighted the emergence of a pathogen that was first identified in Alaska in 2002. Three MLST STs were identified during the 10-year period of 2002–2011; the same 3 STs were recently identified in British Columbia by Shuel, et al. (*13*). Although the first appearance of each ST occurred in a port or major city outside of Region A, outbreaks of disease occurred only in Region A, the region that reported the highest rate of childhood respiratory hospitalizations in the state and the highest rate of Hib disease in the prevaccine era (*3*). Patterns of spread of these Hia STs showed a tendency of slow expansion within a limited geographic region over a period of years. These data suggest several directions for future research and prevention activities.

Extensive evaluation of isolates of invasive Hi disease (1983–2011), and a large study of *H. influenzae* throat carriage in 1982 (*14*) revealed no confirmed isolates of Hia in Alaska before 2002. Reports of Hia carriage in children in Alaska during the early 1980s (*14*) and invasive Hia disease before 2002 (*3*) were incorrect; subsequent MLST evaluation of these isolates and confirmatory re-evaluation by serotyping determined that they were either nontypeable *H. influenzae* or encapsulated *H. influenzae* of other serotypes. Suboptimal accuracy of serotyping by slide agglutination alone for identification of capsular types in general (*15*) and Hia in particular (*16*) has recently been well characterized and likely explains the original false-positive Hia results.

Hia was not identified in Alaska until 2002 and has since increased and caused outbreaks. Although it is possible that Hia existed in Alaska before 2002, it is unlikely that it was commonly circulating during the 1980s and 1990s. The primary focus of the surveillance and carriage studies in those decades was Hib, and thus, surveillance may have missed an occasional Hia case. Serotyping for non–type b encapsulated strains was routinely performed on available *H. influenzae* isolates in Alaska from 1985 onward, and multiple isolates of nontypeable *H. influenzae*, Hid, Hie, and Hif were identified over the 32 years covered in this report. The subsequent rapid expansion throughout the rest of the study period also suggests recent introduction, as opposed to the uncovering of a previously prevalent strain, as apparently occurred for Hia disease in the American Indian population in the American Southwest after introduction of the Hib conjugate vaccine (*17*).

The epidemiologic pattern of invasive Hia disease in Alaska differs strikingly by geographic region. Single episodes of invasive disease caused by each of the 3 Hia STs were initially identified outside of Region A; later appeared within Region A, where they proliferated and spread. Although disease outside of Region A remained rare, within Region A, invasive Hia disease caused by 2 of the 3 ST groups expanded slowly, but persistently, to contiguous geographic areas. In the central part of Region A, rates of Hia disease during the 2009–2011 outbreak among children <5 years of age approached those of prevaccine invasive Hib disease in Alaska (250/100,000) (*3*).

The disparity in rates of disease by geographic region may be partly explained by several factors that are known to contribute to the elevated risk for respiratory infection in general in Region A. Previous studies have shown associations between the rate of respiratory infections and household crowding, decreased availability of in-home piped water service, indoor smoke, and poverty (*18*,*19*). Each of these factors could play a role in facilitating transmission or invasiveness of Hia disease in human populations. These same factors likely play a role in other populations for whom high rates of Hia disease are routinely described, including residents of the American Southwest (*17*) and northwestern Ontario, Canada (*5*, *6**)*, although the factors contributing to increased rates of disease in Utah are less clear (*7*). Recently, invasive Hia cases have also been identified in British Columbia and Manitoba, Canada (*13**, **20*). In contrast, Hia is not a major cause of invasive *H. influenzae* disease in other areas of Canada, the United States, and Europe, where these risk factors are not as prevalent and Hif plays a more prominent role (*21**–**23*). It should also be noted that in the other areas of Alaska, where the above-mentioned contributory factors are less prevalent, the rate of invasive Hia disease between 2002 and 2011 (0.08/100,000) was similar to previously noted rates in the rest of the United States before vaccine introduction (0.04/100,000) (*24*).

The clinical syndrome associated with Hia infection in Alaska from 2002–2011 was similar to that described in Canada, Utah, and the American Southwest (*5**,**7**,**17*,*20*): most cases occurred in children <2 years of age, 84% of case-patients were hospitalized, and the case-fatality ratio was 9%. Of the 13 Alaska Native children infected during the 2009–2011 outbreak, 6 (46%) had meningitis, 1 (8%) child died, and 1 child had serious neurologic complications following a stroke the child experienced in the hospital. Although clinical disease associated with Hia was often severe, the Hia isolates from this outbreak in Alaska do not exhibit the IS*1016*-bexA partial deletion, a characteristic classically associated with more severe disease (*12**,**25*).

Hia infection in infants causes a serious disease with severe sequelae. The severity of disease and increasing rates in recent years have increased interest in identifying prevention and control options. A promising Hia conjugate vaccine, modeled after the Hib conjugate vaccines, has been suggested for control of invasive Hia disease (*4*). However, the population at risk for high rates of Hia disease is so small that further development of this potentially useful tool has not progressed. The presumptive ecologic and transmission characteristics of Hia disease, which rely heavily on analogies with invasive Hib disease, raise the possibility that interrupting person-to-person transmission through chemoprophylaxis may be effective. Current clinical practice in Region A often includes chemoprophylaxis of close and household contacts of Hia case-patients (*26*). However, to this point, there are no reports of secondary invasive disease cases (i.e., a case of invasive Hia disease occurring in a close or household contact of a case-patient with Hia disease), and thus, a key component of the justification for a chemoprophylaxis control strategy is missing: identification of those at risk for secondary disease once a sentinel case has occurred. Finally, several identified risk factors for increased respiratory disease are modifiable, and continuing efforts are underway to address issues of lack of running water, housing ventilation, indoor wood smoke, and other factors, in the high risk area, which may lead to reduced rates of Hia disease. To better define carriage and transmission patterns and further clarify risk factors for invasive Hia disease, AIP is currently evaluating risk factors for disease and Hia carriage among contacts and noncontacts of case-patients in Alaska. These data may contribute to future recommendations for prevention and control of Hia disease in this environment.

The primary limitations of this study are a result of the changing laboratory and surveillance methodologies used over the past 3 decades. Although statewide surveillance of invasive *H. influenzae* disease began in 1980, it was primarily focused on Hib disease, and compliance with the program expanded gradually. We cannot, therefore, confirm that all invasive *H. influenzae* isolates from the early years of surveillance were sent to AIP, or that there was not preferential selection of Hib strains for surveillance. However, by 1983, both nontypeable and non-b encapsulated strains were being received and identified routinely in the AIP laboratory. These isolates were retained, and all Hia strains were subsequently evaluated by MLST by AIP laboratory personnel to confirm strain identity. Although earlier serotyping results suggested the presence of invasive Hia disease in the mid-1990s (and Hia in carriage studies from the early 1980s), subsequent molecular testing and re-serotyping resulted in reclassification of all of these isolates, mostly to nontypeable *H. influenzae*. This experience illustrates the importance of molecular methods in evaluation of epidemiologic patterns, inter-laboratory quality control (*27*), and the importance of close collaboration between laboratory and epidemiology groups.

We confirmed the presence of Hia in Alaska in 2002, and its subsequent emergence as a cause of invasive bacterial disease in an area in rural Alaska associated with high risk for the disease. Its pattern of spread is consistent with slow, person-to-person transmission, with persistence in the high-risk area. Continued surveillance is needed to monitor for outbreaks and galvanize meaningful control efforts. Studies currently in progress in Alaska and other populations should help shape those efforts by providing additional information on risk factors and disease transmission.
